# Role of Tertiary Lymphoid Structures (TLS) in Anti-Tumor Immunity: Potential Tumor-Induced Cytokines/Chemokines that Regulate TLS Formation in Epithelial-Derived Cancers

**DOI:** 10.3390/cancers6020969

**Published:** 2014-04-23

**Authors:** Erica M. Pimenta, Betsy J. Barnes

**Affiliations:** 1 Rutgers Biomedical and Health Sciences, New Jersey Medical School-Cancer Center, Newark, NJ 07103, USA; E-Mail: pimentem@njms.rutgers.edu; 2 Department of Biochemistry and Molecular Biology, Rutgers Biomedical and Health Sciences, New Jersey Medical School-Cancer Center, Newark, NJ 07103, USA

**Keywords:** tertiary lymphoid structure, CXCL13, germinal center, anti-tumor immunity, humoral immunity, B cells, T cells

## Abstract

Following the successes of monoclonal antibody immunotherapies (trastuzumab (Herceptin^®^) and rituximab (Rituxan^®^)) and the first approved cancer vaccine, Provenge^®^ (sipuleucel-T), investigations into the immune system and how it can be modified by a tumor has become an exciting and promising new field of cancer research. Dozens of clinical trials for new antibodies, cancer and adjuvant vaccines, and autologous T and dendritic cell transfers are ongoing in hopes of identifying ways to re-awaken the immune system and force an anti-tumor response. To date, however, few consistent, reproducible, or clinically-relevant effects have been shown using vaccine or autologous cell transfers due in part to the fact that the immunosuppressive mechanisms of the tumor have not been overcome. Much of the research focus has been on re-activating or priming cytotoxic T cells to recognize tumor, in some cases completely disregarding the potential roles that B cells play in immune surveillance or how a solid tumor should be treated to maximize immunogenicity. Here, we will summarize what is currently known about the induction or evasion of humoral immunity via tumor-induced cytokine/chemokine expression and how formation of tertiary lymphoid structures (TLS) within the tumor microenvironment may be used to enhance immunotherapy response.

## 1. Introduction

In order to become an invasive cancer, a tumor must be able to control its microenvironment. Genetic dysregulation, common to all cancers, has implications that reach far beyond the tumor cell. Secreted proteins, cytokines and chemokines affect neighboring cell populations which may then enable angiogenesis, degradation of the basement membrane and evasion of an anti-tumor immune response. It is the ability of the tumor to orchestrate a permissive environment that allows for tumor growth and metastasis. Here we will focus on how epithelial-derived tumors evade the immune response specifically through the dysregulation of specific cytokines/chemokines that regulate the formation of ectopic lymph nodes. It is important to note that while each chemokine has individual functions, it is their action in concert that manifests either an immunogenic or immunosuppressive environment. The relationship between tumor and tumor infiltrating lymphocytes (TIL) is also complex because tumor-derived cytokines influence the expression of TIL-derived cytokines and vice versa. In this review, we highlight the cytokines/chemokines required for ectopic lymph node formation and their role in several cancer types.

We focus on epithelial-derived cancers because much is lacking with regard to our understanding of how epithelial cells-the first barrier we have against pathogen-are able to induce an immune response via cytokine/chemokine secretion resulting in a reprogramming of the tumor immune microenvironment. Their ability to express pro-inflammatory cytokines/chemokines is poorly understood but vitally important to our understanding of how a tumor evades these mechanisms.

Ectopic lymph nodes, or tertiary lymphoid structures (TLS), are extremely important to the formation of both a humoral and cell-mediated immune response. Humoral immunity is dependent on B cells producing antibodies to specific antigen. Cell-mediated immunity relies on activated T cells’ cytotoxic effect on “damaged” cells. In ectopic lymph nodes, as in secondary lymph organs (or classical lymph nodes), the presentation of antigen occurs to both B and T cells making this structure an extremely efficient immunological tool. Therefore, the presence of TLS in epithelial cancers may be vital to anti-tumor immunity.

### 1.1. Germinal Centers and Formation of Tertiary Lymphoid Structures (TLS)

**Figure 1:**
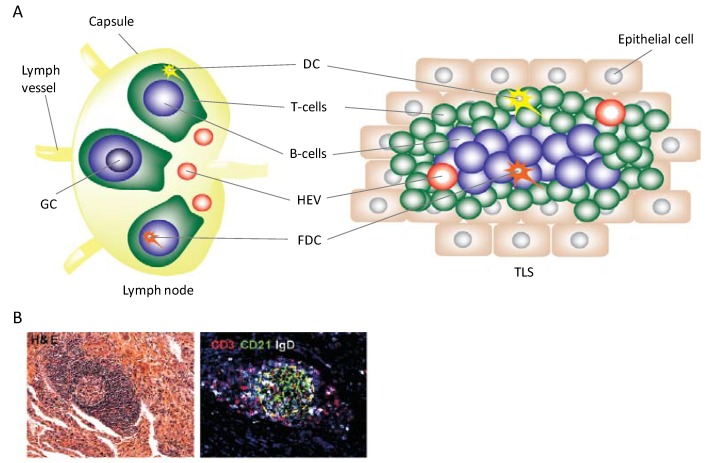
Histological similarities and structural differences between lymph nodes and TLS. (**A**) Both lymph nodes and TLS contain the same cell populations and high endothelial venules (HEV). On the left, a schematic of lymph node structure highlighting B and T cell zones is shown. Each zone contains resident cell populations that upon antigen presentation by follicular dendritic cells (FDC) or DC, and subsequent activation, undergo clonal expansion. Expanded B cell populations form a germinal center (GC). On the right, a TLS schematic showing individual cells aggregating which mimics lymph node histological structure is shown. B cells in this case will also clonally expand and form germinal centers after antigenic stimulation. Structural differences are highlighted; lymph nodes are encapsulated and connected to the lymphatic system via afferent and efferent lymph vessels while a TLS forms within a chronically-inflamed tissue and lymph vessel formation may eventually occur [6]; (**B**) Tissue specimen of TLS structures seen in tuberculosis infection. The left is an H&E stain; the right is an immunoflourescence image staining for CD3^+^ T cells and CD21^+^IgD^+^ B cells [7].

Although not much about the induction of TLS is currently understood, many of the processes that occur during the formation of lymph nodes are mirrored in the development of TLS. TLS formation has mostly been studied in mouse models, but the post-embryonic development of ectopic lymphoid tissue is a commonly-observed phenomenon [1,2,3,4,5]. As illustrated in Figure 1, the same functional cell populations are present in both lymph nodes and TLS but key structural differences occur. TLS are not encapsulated and can be embedded within almost any non-lymphoid tissue [2]. TLS do not form during embryonic development and instead are induced by pathogen or chronic inflammatory signaling [2,4]. 

The first steps in the induction of a TLS are still controversial-lymphoid tissue inducer cells (LTi) may or may not be required as in lymph node development [1,2]. During lymph node development, lymphoid tissue inducer cells (LTi) that originate in the fetal liver express lymphotoxin (LT)α and LTβ and are attracted to LTαβR-expressing mesenchymal cells that organize lymph node formation at pre-determined sites throughout the embryo [1,2]. Additional chemokines attracting LTi cells are CCL19, CCL21 and CXCL13, also expressed by the mesenchyme [1,2]. These chemokines attract the lymphocyte subsets that will reside in the forming lymph node [2]. CCL19 is the ligand for CCR7, a receptor expressed on subsets of T cells and dendritic cells (DCs) [8,9]. CCL21 is highly expressed in high endothelial venules (HEVs)-specialized vessels carrying circulating lymphocytes in and out of the lymph node-and in the T-zone of lymph nodes [10,11,12,13]. CCL21 also signals through the CCR7 receptor on natural killer (NK) cells, naϊve and memory T cells, and DCs to recruit them to developing lymph nodes and aid in their activation and function during an active immune response [10,11,12,14,15]. CXCL13 is one of the 4 most potent B cell chemoattractants known [7], causing an influx of migrating B cells as well as a subset of circulating T cells that express its cognate receptor, CXCR5 [16,17,18,19]. Disruption of any part of this chemokine network will disable the proper formation and function of the lymph node. 

Evidence exists that circulating B, T or dendritic cells (DC) may be able to act in response to chemokines secreted by the injured tissue and take the place/act as LTi cells themselves [1]. For example, after stimulation with CXCL13 or CCL21, LTαβ is expressed by T cells, supporting their role as possible LTi cells [9]. Interestingly, non-classical cytokines may also induce LTαβ expression in T cells such as IL-4, IL-7 and IL-2 [7]. The chemokines necessary for induction of TLS, however, are at least in part identical to those required for lymph node formation [1,2,5,20]. The administration of CCL21, CXCL13 or LTα on their own can induce TLS in mouse models [1,9].

As in lymph node formation, LTαβ expression promotes CXCL13 and CCL21 expression forming a positive feedback loop continuously augmenting the secretion of these critical homing chemokines [2,9,20]. LTα induces the formation of HEVs and the activation of follicular helpter T-cells (Tfh) which may be the circulating counterpart to FDCs. CCL19 and CCL21 signal via the CCR7 receptor to call in and regulate T cells while CXCL13 recruits and activates B cells [1]. The CCL21-dependent recruitment of DC and natural killer (NK) cells from the peripheral circulation may eventually lead to the development of lymphatic vessels in TLS [6]. As described above, the components of TLS are strikingly similar to those in lymph node formation and allow us to infer that TLS can also promote powerful and efficient immune responses.

### 1.2. Epithelial Cell-Induced Immunogenicity

While not classically thought of as immune cells, epithelial cells have a pivotal role in establishing defense against pathogen(s) as they are the first line of defense an offending agent will come into contact with. In addition to serving as a physical barrier to the outside environment, epithelial cells have the capacity to induce an immune response by upregulating potent immunogenic cytokines/chemokines as seen in breast [21,22], colon [23], salivary gland [24], lung [16], Fallopian tube epithelium [25], synovial epithelium [26], and even in the epithelium of the central nervous system (CNS) [7]. 

In response to certain pathogens, evidence shows that the cytokines/chemokines released by epithelium can organize TLS. Most epithelial cells express the LTαβR, indicating that they are likely responsive to LTαβ signaling [27]. Pathogens found to induce the expression of TLS-associated cytokines/chemokines include *Mycobacterium tuberculosis* (Mtb) [1], *Escherichia coli* [21], and the influenza virus [16] among others, indicating that this may be a relatively unexplored but common and powerful immune process induced to protect the host. 

Before granuloma formation occurs in latent Mtb infection, the formation of TLS occurs to increase the chances that B cells and other antigen presenting cells (APC), and T cells will interact and mount an effective immune response [1]. After early infection with Mtb, lung parenchyma (both resident immune cells and non-immune cells) express CXCL13 [16]. The CXCL13-CXCR5 axis is required for B cell entry and organization into TLS [28]. 

The importance of B cells in secondary lymph nodes and even in TLS has been explored for some time, while the role of CD4^+^CXCR5^+^ T cells is less well known. These cells are responsive to CXCL13 by their CXCR5 receptor and they travel to follicles after infection in a CXCL13-dependent manner [29]. These cells basically act as the peripheral version of a Tfh [18]. Tfh are found in already-established primary and secondary lymph organs and are required for successful plasma cell differentiation and subsequent differentiation of memory B cells [19]. Circulating CD4^+^CXCR5^+^ Tfh cells, herein also referred to as Tfh, are necessary for TLS function and have been identified as high expressers of ICOS (inducible T cell co-stimulator, CD278), PD-1 (programmed cell death 1), Bcl-6 and produce IL-21 for germinal center formation [15]. It is now known that both Tfh and B cells must be present to form an organized and functional TLS [19]. 

Once B cells and Tfh are in close proximity within the TLS, exposure to antigen causes those antigen-specific B cells to clonally expand just as a germinal center would in a lymph node [17,30]. This occurs successfully with the secretion of IL-21 and other activating cytokines from Tfh [19]. The CXCL13-CXCR5 axis is extremely important for clonal proliferation because it greatly enhances B cell activation by inducing the gathering of antigen at the B cell membrane to enhance B cell receptor (BCR) signaling [31] thus making these stimulated B cells potent APC [32]. Within the germinal center, Tfh cells induce AID expression in the antigen-specific B cells allowing somatic hypermutation to occur [17,30,33]. Clonal selection for a high-affinity antibody and isotype switching then occurs and finally some B cells become CD19^+^CD20^−^CD138^+^ plasma cells while others become CD27^+^CD38^−^ memory B cells [1,30]. At that point, the TLS is functioning with APC such as DC, clonally expanded B cells stimulated to produce specific antibody with the help of Tfh cells, plasma cells secreting antibody, and memory B cells that will confer long-term immunity.

In addition to the formation of antigen-specific antibodies and memory B cells, activating and anti-apoptotic signals are sent to macrophages [16,34] and high levels of IFN-γ are produced by newly-activated T cells [16]. This illustrates the capacity of TLS to reach far beyond B cells and participate in the activation of the adaptive immune system in a local immune response. In summary, an epithelial cell has the capacity to induce the formation of TLS primarily based on its ability to express CXCL13 and perhaps CCL19 and CCL21 and also respond to LTαβ signaling. These chemokines will attract B and Tfh cells to the area, allowing for the B cells to become efficient APC and begin pathogen-specific antibody production. In addition, other immune cell types become activated, such as macrophages and CD8^+^ T cells, allowing for a full and effective response to pathogen.

The powerful immunogenic capabilities of TLS are exemplified when ectopic lymph nodes are not shut down or controlled effectively and autoimmunity is induced. For example, in Sjögren’s syndrome, the organization of TLS seen in salivary glands is induced in the same way as a TLS response to pathogen (via CXCL13 expression) [24], with autoantibody production occurring in some cases [1,35]. TLS have also been seen in rheumatoid arthritis [24,26], Hashimoto’s thyroiditis, Grave’s disease, *H*. *pylori* infection, myasthenia gravis, multiple sclerosis, systemic lupus etythematous (SLE) and in allograft rejection [5,24]. This evidence suggests that the depletion of auto-reactive B cells may not be as efficient in TLS relative to bone marrow [35]. While the survival of auto-reactive B cells is generally not favorable, auto-reactive antibodies may be useful as part of an anti-tumor immune response.

### 1.3. A Role for TLS in Epithelial-Derived Cancers

A functioning immune system is vital for systemic tumor surveillance on a daily basis. Without proper immune surveillance and response capabilities, cancer is more likely to occur. This is corroborated by the fact that immunosuppressed populations have a higher cancer incidence than the general population [36]. These populations include organ transplant recipients, those undergoing treatment for autoimmune disease, or cancer patients receiving systemic chemotherapy [37]. Organ transplant recipients have a 5%–6% chance of being diagnosed with cancer, usually of an epithelial origin, while those on methotrexate (anti-folate therapy) for arthritis see an increase in leukemia incidence. While the increase in leukemia may be in part due to the chemotherapy itself, it has been postulated that a lack of immune surveillance is also to blame [37].

Therefore, in a person with an otherwise normal immune system, we can expect to see that at least some who get diagnosed with cancer will show signs of mounting an anti-tumor immune response. Indeed, ectopic lymphoid structures/TLS have been documented in lung, colon, breast, ovarian, renal and germ cell cancers, as well as melanoma [17,38,39,40,41,42,43]. Understanding the mechanisms involved in these processes may allow us to augment a host immune response to tumor with the goal of long-term or complete remission.

While TLS have been seen in several tumor types, not every cancer patient will develop them and when they do occur, they vary in functionality. Some tumor types are more likely to induce TLS formation indicating that the tumor itself plays a major role in either the hindrance or initiation of this humoral immune response.

**Figure 2:**
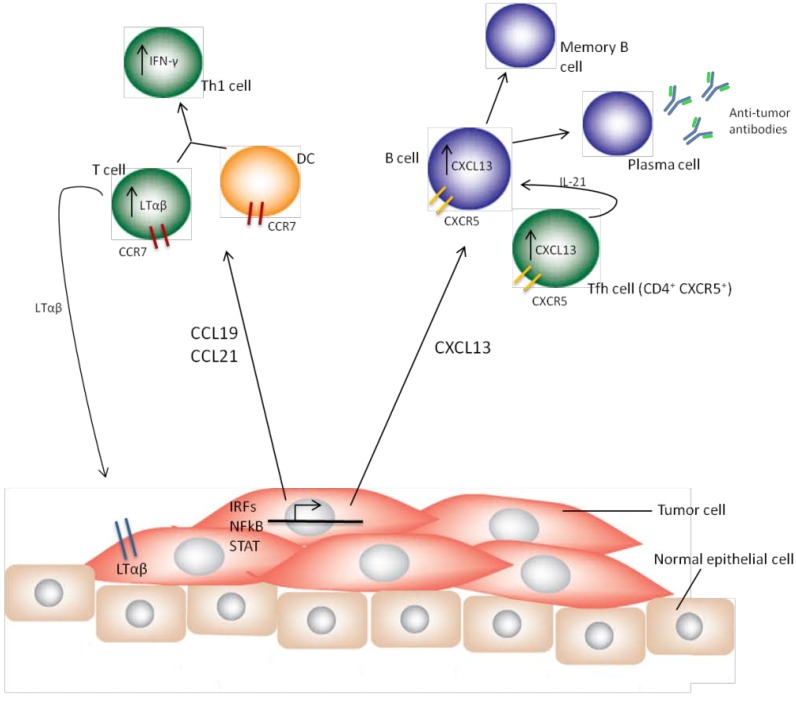
Working model of an IR^+^ tumor and TLS induction. In IR^+^ tumors, expression of transcription factors such as the interferon regulatory factors (IRFs), NF-κB and STAT molecules regulate TLS-inducing cytokines and chemokines. Tumor secretion of CCL19 and CCL21 recruits CCR7^+^ DC and T cells. CCL19 and CCL21 induce LTαβ expression and secretion from T cell populations which may further stimulate inflammatory cytokine release from tumor cells via LTαβR signaling. Tumor-derived CXCL13 recruits B cells and CD4^+^CXCR5^+^ Tfh cells. The Tfh cells stimulate B cell differentiation and activation in part via IL-21. This promotes the development of anti-tumor memory B cells and plasma cells secreting tumor-specific antibodies. With a functional TLS in place, efficient antigen presentation, cell activation and differentiation occurs for both a humoral and cell-mediated anti-tumor immune response. In an IR- tumor, many of the regulatory transcription factors and/or their downstream chemokines are downregulated. In the absence of TLS-inducing chemokines, severe immune deficits occur allowing for tumor immune evasion.

Analysis of the cytokine/chemokine molecular gene signature of some solid tumors offers insight into which cancers and even which particular patients are more likely to have organized TLS via gene profiling. Since it stands to reason that some tumors are more immunogenic and others more immunosuppressive, we and others hypothesize that immunogenic tumors inherently have a better prognosis. There are several ways one can attempt to measure the “immunogenicity” of a tumor; from the DNA/mRNA levels seen in a genetic signature to the number of responsive/activated lymphocytes, or TIL, attracted to the tumor bed. In many solid tumors, all of these measurements have been used and a general consensus reached: immunogenic tumors, with immune response positive (IR^+^) gene signatures and/or increased TIL, have a better prognosis [35,44,45,46]. Figure 2 illustrates a working model of how IR^+^ tumors may be able to induce TLS formation. The following sections will summarize how immunogenicity has been studied in several solid cancer types and the implications for TLS formation.

#### 1.3.1. Breast Cancer

Breast cancer is traditionally thought to be a very immunosuppressive tumor type. Increased immunogenicity has most commonly been measured via lymphocytic or immune cell infiltrate. A study by Denkert *et al*. analyzed 1058 tumor samples by immunohistochemistry (IHC) and microarray and found that 676 of those samples could be identified as having either a good or bad prognosis based on several factors [44], one of which was immune cell infiltrate: the more TIL, the better the prognosis [35,44]. Alexe *et al*. mirrors these results in Her2/neu positive breast cancers [46]. With a 99 month overall survival and 11% recurrence rate, Her2/neu positive breast tumors expressing high levels of lymphocyte-associated genes fared much better than tumors with low levels (33 month survival, 33% recurrence rate) [46]. The typical TIL populations found in most breast cancers are T cells (60%–90%, mostly CD4^+^), B cells (about 20% or less), monocytes (less than 10%), and NK cells (less than 5%) [39,45].

With regard to infiltrating T cells, an increased presence of CD8^+^ T cells has long been accepted as a positive prognostic indicator via their ability to produce IFN-γ [39,47] as they have the ability to function as cytotoxic T cells. The role of CD4^+^ T cells is more complex. Their particular role in tumor progression or regression may be extremely dependent on the immune microenvironment. In extensively infiltrated tumors, CD4^+^ T cells have been shown to be antigen-experienced and necessary for the function of CD8^+^ T cells so much so that even increased CD4^+^ T cells have been associated with a better prognosis [39]. IL-17-producing effector T helper (Th17) cells, generally thought to be pro-inflammatory, may be even more “context dependent.” They may synergize with IFN-γ to augment anti-tumor immunity [39] but their role is complex and has not yet been well characterized. Other CD4^+^ T cells, as will be discussed below, are required to bridge the gap between cell-mediated and humoral immune responses. 

While most breast cancers have CD4^+^ T cells as their dominant TIL, approximately 20%–25% have B cells as the major immune cell population [30,48]. These patients, based on their B cell infiltrate alone (independent of the CD8^+^ T cell infiltrate), have a better prognosis [49,50,51]. Specifically, Mahmoud *et al*. examined 1470 tissue samples for CD20^+^ cells and saw increased survival and a longer disease free interval [49].

This powerful positive prognostic evidence illustrates that a B cell-mediated anti-tumor immune response may occur. B cells may even be among some of the first responders, as B cells can aggregate before breast disease becomes invasive [30,32]. Medullary breast cancer, famous for its intense TIL and in particular B cell infiltrate, has an 84% 10-year survival rate compared to 63% in non-medullary breast cancers [51]. A small study by Nzula *et al*. examined primary breast tumor samples for the presence of B cell infiltrate; importantly, the patients had not yet undergone any treatment [45]. The significance of this is that non-specific chemotherapy agents are notoriously immunosuppressive and will have an impact on the host immune response. In the primary tumors, Nzula *et al*. found a direct correlation between B cell infiltrate and improved prognosis and that B cells present in the individual patients showed evidence of antigenic stimulation [45]. Genetic analysis of the B cell populations was performed on microdissected B cells from the tumor rather than whole tumor isolates thus reducing the possibility of contaminating genomes from other tumor-associated cells. Results from this study indicated that V(D)J recombination events had occurred, as well as clonal proliferation [45,51]. Even more striking was the finding that independent individuals had similar V(D)J rearrangements, indicating that there may be a common, non-random antigen present on some breast cancers [45].

Others have shown evidence of mature antibody responses by TIL B cells. In addition to V(D)J rearrangements, class-switching occurred from IgA, found in normal breast tissue, to IgG1 and IgG2 [35,48,51,52]. Pavoni *et al*. showed that when a B cell-mediated immune response could be observed in a breast cancer, up to 70% of B cells present were part of a clonal expansion group. No IgG secretion or oligoclonal cells were found in normal tissues [52].

V(D)J rearrangement, class switching, and clonal expansion are only useful if they result in functional and selective antibodies. In about 50% of breast cancer patients, antibodies against known breast tumor antigen are detectable [35,53]. Some of the most common host-derived antibodies target Her2, p53, MUC1, and endostatin [53], and to date over 250 breast cancer antigens have been identified [35]. Non-identified antigens, also known as “cryptic epitopes”, which were discovered by sequencing V(D)J regions and not finding a matched antigen, have also been documented [51,54] and are specific for binding to breast cancer cells and not normal tissue [54]. Together, these data demonstrate that active, humoral immune responses do occur in at least some breast cancer patients although, these processes are not solely B cell dependent. B cells require cell-dependent and cytokine-dependent activation and regulation in order to complete these complex tasks. B cells must form structures that increase the efficiency of antigen presentation and T cell activation. In short, these B cells must form TLS in or near the tumor site. Further evidence of functional TLS formation in breast cancer is the presence of HEV in breast cancer that associates with a better prognosis possibly due to the observed increase in B and T cell infiltrate [55].

As mentioned earlier, other CD4^+^ T cells may be important for B cell activation and autoantibody production within the tumor microenvironment such as CD4^+^CXCR5^+^ Tfh cells. Coronella *et al*. documented that B cells aggregate in “lymph node like” germinal centers at tumor margins in which oligoclonal expansion is observed [48]. Furthermore, Gu-Trantien *et al*. characterized the presence and role of Tfh cells found at TLS in breast cancer [39]. The study took 20 untreated breast cancer samples, non-enzymatically dissociated the tissue and isolated Tfh for analysis. Before dissociation, however, histological examination revealed extensively-infiltrated tumors with TLS present near the edges of the tumor bed whereas minimally infiltrated tumors did not commonly have TLS. After expression analysis of the Tfh cells isolated from heavily-infiltrated tumors, they found that these Tfh were quite similar to traditional Tfh found in secondary lymph organs. The heavily-infiltrated tumor Tfh cells expressed more activation markers, including CD200, CXCL13, ICOS and PDC1, compared to Tfh cells isolated for tumors with low levels of immune infiltrate [39]. Somewhat expectedly, they also found that tumors with lots of TIL had more active CD8^+^ T cells, confirmed in part by elevated IFN-γ expression [39].

Importantly, CXCL13 was found to be the most sustained chemokine expressed, not decreasing dramatically even after 24 hours in culture without stimulation outside of the tumor. In contrast, IFN-γ levels quickly dropped to unstimulated levels [39]. This points to a pivotal and expected role for CXCL13 as one of the major organizers of an anti-tumor immune response in TLS. In addition, expression of CXCL13 correlated with immune infiltrate, a strong Th1 cell presence and the formation of TLS. The presence of CXCL13-producing Tfh cells or just CXCL13 alone was better at predicting clinical responses regardless of Her2 or triple negative subtype [39]. Thus, as suggested from gene signature studies, CXCL13 was the most predictive marker for prognosis, and even more reliable than Th1 signatures for survival [39]. Some controversy exists as to whether or not CXCL13 is produced by the tumor cells themselves [22,39,56]. Panse *et al*. saw an increase of CXCL13 in serum samples and tumor samples of breast cancer patients; however, they did not microdissect to confirm the cells responsible for this expression [22]. They concluded from their IHC data that the tumor was not the primary source of CXCL13. Gu-Tratien *et al*. found that the CD4^+^ T cell infiltrates were most responsible for CXCL13 expression [39]. Data from our lab suggests that the tumor is in fact capable of producing CXCL13 in some cases [57]. This may be in agreement with Gu-Tratien *et al*. since a small amount of tumor-derived CXCL13 may attract the B and T cells that will subsequently produce much more of this potent chemokine [39]. Biswas *et al*. has implicated the CXCL13-CXCR5 axis in increasing the expression of mesenchymal markers such as vimentin, N-cadherin, Snail, Slug and MMP-9 [58]. While they evaluated this phenomenon in both MDA-MB-231 and T47D cells, which have already gone through EMT, it would be interesting to see the effect of CXCL13 on cell lines that have not yet undergone this transition [58]. 

While CXCL13 in breast cancer has been the most extensively studied to date, the expression of other TLS-inducing chemokines has also been implicated in this disease. When MCF-7 breast cancer cells were made to express high levels of CCL21, increased tumor immunogenicity was noted via HLA and TAP-1 expression increases. Xenograft mouse models using MCF-7 cells expressing or not expressing CCL21 show that in the presence of CCL21, tumor growth is inhibited and T cell activation is enhanced [59]. Conversely, Kim *et al*. analyzed 15 patient samples to assess CCR7 and CCL21 expression levels and found that they were both increased in the tumor when compared to normal [60]. Blocking the autocrine signaling between ligand and receptor inhibited cell movement. Muller *et al*. also found that CCR7 was upregulated in human breast tumor tissue (*n =* 12) compared to normal (*n =* 5) and hypothesized that high CCL21 expression in lymph nodes may then attract the CCR7 positive tumor cells [61]. The relatively small sample size and heterogeneity of the tumor types however, may not be an accurate look into the overall picture of CCL21 expression in breast cancer. For example, if these were early cancers, perhaps CCL21 expression was upregulated in an attempt to mount an immune response then later diminished to ensure tumor survival. 

CCL19 has a more complex role in breast cancer, being used successfully as an adjuvant in cancer vaccines [62,63] but also is implicated in lymphogenous tumor metastasis [8]. Cassier *et al*. analyzed breast tumor samples before patients underwent treatment and found that about half of the tumors expressed CCL19. Furthermore, the presence of infiltrating CCL19-expressing DC correlated with an increased risk of relapse which may implicate CCL19 in metastasis via the lymphatic vessels [8]. However, when administered exogenously both intratumorally or intradermally alongside a Her2/neu DNA plasmid vaccine, CCL19 was able to elicit a Th1 anti-tumor response in a mouse model of Her2/neu positive breast cancer [62,63]. 47 days post-tumor xenograft injection, 58% of mice given CCL19 and the Her2/neu adjuvant vaccine were still alive compared to only 22% of mice given the Her2/neu plasmid vaccine alone [62]. 

In summary, data support that the presence of B, Th1 and Tfh cells within TIL are extremely good prognostic cellular markers since these three cell types work in concert to produce both cellular-mediated and humoral anti-tumor immune responses. Most striking, however, is the high prognostic power of CXCL13 expression across breast cancer subtypes even in triple negative and Her^2+^ tumors. It seems plausible that CXCL13 is the main orchestrator and organizer of TLS. From its ability to recruit circulating Tfh and B cells to the site, to increasing the efficiency of antigen presentation and B cell activation, this chemokine and its expression by tumor cells is essential for the formation of an anti-tumor immune response in breast cancer. 

#### 1.3.2. Colon Cancer

The immune environment of the colon is markedly different than that of the mammary duct. The colon is constantly exposed to foreign antigen which under healthy conditions (non-autoimmune) does not elicit an inflammatory response. Surprisingly, even in this relatively tolerant tissue, an anti-tumor response can be mounted and similarly to breast cancer, colon cancers can be stratified into IR^+^ or negative (IR-) tumors [40,64]. In particular, Coppola *et al*. did a metagene analysis with 326 cancer specimens and 21 normal and narrowed in on 12 chemokines that correlate with the presence of TIL and increased survival [40]. As expected, CXCL13 was included as one of the prognostic genes as well as CCL19, among others [40]. Expression of these genes in the IR^+^ colon cancers is associated with increased survival, independent of tumor stage, previous treatment or microsatellite instability [40]. A study by Mumtaz *et al*. also showed that colon cancer tissue specimens from 74 patients had lower expression of CCL21, further diminishing their ability to elicit an immune response [11]. As is the case in breast cancer, several immune cell populations constitute TLS in colon cancer. In general, a high density of TIL is a more accurate predictor of increased survival than traditional tumors/nodes/metastases (TNM) staging [65,66,67]. Of interest, colon cancers with microsatellite instability usually have more TIL [65]. This may be due to an increased mutational burden leading to many more non-self antigens [65]. 

T cell infiltrate no doubt plays a role in colon cancer, with high CD3^+^ and CD8^+^ signatures consistent with a good prognosis [65,68]. Th1 expression markers like interferon regulatory factor 1 (IRF1) were also good prognostic indicators [66]. Immunosuppressive Tregs have been given some attention in colon cancer, conferring a worse prognosis when present without CD8^+^ T cells [68]. 

Evidence of increased B cell activation in colon cancer patients exists via increased Toll-like receptor (TLR) signaling in peripheral B cells [69] and tumor-specific antibody production [70] as seen in breast cancer. Mouse models of colon cancer demonstrate the capacity for TLS to form. In a colitis-associated colon cancer model, TLS were analyzed and found to contain the expected aggregation of FDC, B cells, T cells and HEV [38]. While these aggregates were also observed during inflammatory colitis, B cell proliferation within follicles was noted only after polyps became malignant growths [38]. This may indicate that only after a colon cancer becomes invasive is an immunogenic threshold met, but it also points to the interesting possibility that the presence of TLS may have played a role in malignant development. Although most evidence demonstrates otherwise, this is not a possibility we can ignore as there is no definitive answer as to whether the malignancy or TLS formation occurred first. Using human ulcerative colitis tissue, Carlsen *et al*. saw that 100% of samples had expression of CXCL13 and every B cell and a portion of the T cell infiltrate expressed CXCR5 [23]. Kirman *et al*. showed that mice with colon cancer burdens produced tumor-specific antibodies [70].

Human studies mirror those discussed above. Examples of TLS in colon cancer but not in normal tissue have been found [38,40] and shown to contain classic TLS cell populations such as CD21^+^ FDC [38]. B cells and other TIL residing in 11 independent colon cancers were EBV-immortalized for subsequent study [71]. These cells were found to be CD23^+^, a sign of antigen exposure or maturation, formed clonal populations, had undergone somatic hypermutation and class switch recombination so that IgM, IgA and IgG were produced and were specific to tumor antigen [71]. It must be pointed out that the process of EBV-transformation may have influenced the cell markers and behavior observed, but coupled with our current knowledge of TIL B cells in colon cancer, it seems likely that this particular B cell activation is in fact tumor-specific. Maletzki *et al*. further showed that these B cells express high levels of major histocompatibility class I (MHC-I), -II and CD80, indicating that they may also be acting as efficient APC in colon cancer [71]. 

CCL19, as in breast cancer, slowed tumor growth in a murine colon cancer model while increasing the influx of DC and T cells to the tumor site [72]. A later study of human TLS present within tumors showed that high expression of CCL19 in resident DC allowed for greater CD8^+^ T cell expansion and an increase in granzyme B expression, one of CD8^+^ T cell’s methods of cytotoxicity [73]. Thus, it seems likely that TLS formation in colon cancer is similar to that seen in response to pathogen and in breast cancer and may also confer a good prognosis.

Recently, Di Caro *et al*. investigated the prognostic value of TLS in colorectal cancer by following 351 stage II or III colon cancer patients with no clinical signs of metastasis to correlate TLS and TIL with disease progression and survival [74]. In patients that ended the study with less metastasis and a better prognosis, highly vascularized TLS (*i*.*e*., TLS with a high density of HEV) were present. The presence of TLS also correlated with more CD3^+^ T cell infiltrate [74]. This confirms the importance of TLS as a possible marker for better prognosis in colon cancer and implies that a more effective anti-tumor response may occur in tumors with well-organized TLS.

#### 1.3.3. Melanoma

The stratification of classically immunogenic melanomas into IR^+^ and IR^−^ groups has also been done. Gene profiling of human melanomas identified CXCL13 and IL-8 as components of a smaller group of 12 genes found to be diagnostic markers from a larger 200-gene signature [56]. In a similar manner, Jonsson *et al*. took 57 stage IV melanoma biopsies before treatment and used gene expression profiling to further stratify these tumors into 4 subtypes: IR^+^, pigmentation differentiated, proliferative and stromal gene expression [75]. Interestingly, the IR^+^ group showed upregulated expression of pro-inflammatory *IFNGR10* and *CXCL12*; low expression of these genes conferred poorer outcomes. In fact, by stratifying all biopsies into either IR^+^ or IR^−^ groups, the IR^+^ group mean survival was 55 weeks and IR- group was 18 weeks [75]. Furthermore, the IR^+^ group had dense lymphocytic infiltrate made up of mostly T cells but always having a B cell component present as well. The authors noted that IR^+^ tumors had many more gain of function mutations than deletions [75]. Additional studies by Messina *et al*. analyzed over 14,000 solid tumors and found that the expression of 12 chemokines in particular were indicative of an overall better prognosis in melanoma and presence of TLS [41]. Among the 12 are CCL19, CCL21 and CXCL13 [41]. These data offer insight into how normal tissue can readily respond to tumors and how loss of these “alarm signal” chemokines allows for immune evasion. Data suggest that tumors lacking these deletions may retain their ability to elicit an immune response, therefore conferring a better prognosis. Another possible immune signal that may be lost during melanoma development is CCL21. Forced expression of CCL21 in melanoma cells caused an increase of NK and CD8^+^ T cell infiltrate resulting in a bolstered immune response when compared to melanomas lacking CCL21 [12]. 

CCL19 remains more complex, and as with breast cancer, may be implicated in the spread of melanoma to neighboring lymph nodes through the CCR7-CCL19 axis [76]. Dobner *et al*. measured CCR7 expression in 70 human melanoma patients and found that it is consistently expressed and correlates with liver metastasis which they hypothesize occurs through lymphogenous spread [76]. However, the CCR7-CCL19 signaling pathway may not be all bad; evidence exists that CCR7 can bring antibodies into endosomes for potential CCL19-conjugated treatment strategies [77].

LTαβR is expressed in most melanoma cases [27,78,79]. Exploiting this signaling network, Schrama *et al*. conjugated LTα to a tumor-specific antibody in a melanoma mouse model. When given quickly after tumor injection, this treatment complex completely inhibited growth in 75% of mice and increased survival time dramatically [79]. Formation of TLS and clonally expanded, active T cells were observed at the tumor site of these animals [79]. Interestingly, this effect is diminished if the LTα-antibody complex is administered later than 10 days post xenograft. This may indicate that the tumor has already established a powerful immunosuppressive environment or that the tumor burden is too great to overcome the simple treatment at that time. 

A brief look at work by Wang *et al*. confirms the presence of an active T cell response in melanoma [80]. Using fine needle aspirates from 25 patients who were under standard treatment for melanoma (including IFN-α), they performed gene analysis and saw that markers of an active T cell response, such as IRF1, IRF2 and TLA-1, were present in lesions that responded to treatment. They also observed an increase in EBI3 (Epstein-Barr virus induced gene-3) which induces IL-12 expression and is associated with APC. While it is currently unclear which cell population(s) is responsible for the observed gene expression since total tumor tissue was analyzed, evidence of an active immune response present after IFN-α treatment in responding lesions is likely to be T cell-dependent [80].

It may be the case that in melanoma a T cell response is more effective or is the more common result of tumor-specific initiation. However, this does not mean that B cells and possibly TLS do not play a role as well. To examine the role of B cells in melanoma, DiLillo *et al*. used a syngeneic mouse model of melanoma and depleted B cell populations with anti-CD20 antibody [81]. Depletion of B cells in this manner allowed for a normal immune system and examination of whether B cell loss alters tumor formation after injection of the B16 melanoma cell line. At both 7 and 14 days post-tumor injection, twice the number of tumors were found in the anti-CD20 treated cohort [81]. The B cell depletion didn’t affect the ability of T cells to migrate and survive within lymph nodes but did inhibit T cell proliferation due to antigen stimulation, specifically in the CD8^+^ T cell population. Anti-CD20 treated mice had 45% less IFN-γ, TNF-α and CD4^+^ T cells in their draining lymph nodes relative to control mice [81]. These data support that B cells are critical for a functional T cell response in melanoma.

Returning to human melanoma, analysis of a panel of 106 melanoma tissue samples revealed that about 26% had B cell aggregates that correlated with the presence of activated T cells [82]. Metastatic lesions had consistently less B cells than non-metastatic primary tumors. A 78% 5-year survival was observed in patients with high B cell density *vs*. 59% in those with low B cell infiltrate. Interestingly, B cell aggregation did not correlate any stronger to survival than B cell infiltrate alone [82]. 

In a more specific look at TLS formation in melanoma, Cipponi *et al*. took 29 metastatic skin lesions and analyzed them for B cell and TLS content [17]. 14 of the 29 tumors had CD20^+^ aggregates, 10 of 29 had both B cells and FDC aggregates, and 7 of 29 had complete TLS, including follicle formation staining positive for Ki67, AID and the presence of HEV and T cells. The TLS were always in direct contact with tumor cells. In those primary tumors that resulted in visceral metastasis, no complete or functional TLS could be observed [17].

While it currently seems likely that fledgling immune responses to melanoma may be T cell dominant, there is increasing evidence that B cell function is required for T cell activation and that the formation of TLS in melanoma is beneficial. The formation of TLS in melanoma is likely to be CXCL13-mediated, as in breast and colon cancer, although melanoma immune response literature is currently T cell-focused.

#### 1.3.4. Lung Cancer

The importance of tumor immune signatures across all lung cancer subsets was highlighted by Rohrbeck *et al*. showing that adenocarcinoma, squamous cell carcinoma and small cell lung cancers all had dramatic decreases in the expression of immune-regulatory genes [83]. With regard to non-small cell lung cancers (NSCLC), others have found that immune response genes are the most dysregulated subset of genes [84,85] and that higher expression of immune response genes predicts both recurrence-free and overall survival [86].

In human NSCLC, 35% of 91 tumors stained for Bcl-6 and CD21, which together indicate the presence of proliferating B cells within a follicle of a TLS both intratumorally and on the tumor margins [42]. Stage I NSCLC had the highest frequency of germinal center formation. De Chaisemartin *et al*. did a retrospective study on 75 NSCLC tissues compared to 5 healthy lung biopsy tissue samples and found that an increased density of DC was prognostic; 90% survival at 40 months compared to 50% with low DC TIL density [87]. While the densities of B or T cells did not directly correlate with survival, their presence was increased in the tumors with high DC infiltrate. It is important to note that their aim was to look at general populations so the staining was performed with antibodies recognizing CD3, CD4, and CD8 for T cell subsets, CD20 for general B cells and CD21 for FDC [42], therefore analysis of specific TLS populations was not possible. In addition to observing TLS within NSCLC and detecting CCL19, CCL21, CXCL13, CCL17, CCL22 and IL-16 expression, T cells within TLS were found to express significantly higher levels of the receptors for these TLS-associated cytokines/chemokines suggesting recruitment of the T cells to the tumor and formation of an active, functional germinal center [87]. 

CCL19 was injected into mice with lung tumor burdens in two studies [88,89]. Intranodal injection of CCL19 in a bronchoalveolar cell carcinoma mouse model caused an increase in T cell and DC infiltrate and also seemed to have a systemic immune effect. Splenic lymphocytes in the CCL19-injected animals showed higher levels of IFN-γ and the anti-angiogenic chemokines CXCL9 and CXCL10 [88]. Intratumoral injection of both CCL19 and IL-7 slowed tumor growth and completely eradicated lung tumors in 5/6 mice [89]. However, as seen in other cancer types, increased risk of tumor cell migration might also occur with CCL19 administration. Zhang *et al*. showed that incubating the A549 lung cancer cell line with CCL19 caused increased expression of heparanase which may, along with the CCR7-CCL19 axis, facilitate cell migration and metastasis [90]. The exact mechanism should be tested in various cell lines and confirmed *in vivo* but there seems to be a trend across cancer types that CCL19 may potentially drive metastasis.

Two murine studies used adenoviral vectors to express CCL21 in DC populations and both reported that increased CCL21 levels caused an increase in lymphocyte migration to tumor [14,91]. Kar *et al*. specifically showed that in addition to an increase in T cell migration, lung tumor growth was inhibited, an increase in antigen presentation was observed and antitumor immunity was enhanced [14]. A third study introduced CCL21 protein at the tumor site and found that these tumors had reduced angiogenic activity and increased T cell activation indicated by high IFN-γ, CXCL9, and CXCL10 levels [88]. 

DC infiltrate on its own is a positive prognostic marker in NSCLC as well as in colorectal carcinoma and renal cell carcinoma [92,93]. Goc *et al*. found that TLS-associated DC populations correlated significantly with CD8^+^ T cell infiltrate in NSCLC. After analyzing 458 NSCLC specimens for TLS, DC, and CD8^+^ T cell densities, they found that the presence of TLS-associated DC and CD8^+^ T cells was a strong, positive, prognostic indicator for overall survival [94]. In mice, T cell activation was shown to induce tumor rejection in a mechanism involving NF-κB [95] and in humans, a high density of TLS was indicative of long-term survival [87]. Lohr *et al*. showed after microarray analysis of 355 NSCLC cases that the presence of CD138^+^ plasma cells conferred an 80% 2-year survival *vs*. 70% with low CD138^+^ infiltrate [96]. In a smaller study, one of 7 patients with NSCLC that had TLS was still alive at 24 months post-study while 8/36 patients without TLS had metastasis; 1 died at 18 months post-study [42]. Admittedly, the sample size is small, but is in agreement with the data presented above that TLS formation in lung cancer, most explored in NSCLC, is generally a positive prognostic indicator and involves the presence of plasma B cells, DC and T cell activation.

#### 1.3.5. Pancreatic, Cervical, Ovarian, Oral Squamous Cell and Gastric Cancers

Evidence of humoral immune responses also exist for cancers that are typically hard to treat and with a poor prognosis. Serum antibodies to tumor-specific antigens have been documented in pancreatic, cervical, gastric and ovarian cancers [35,39,52,54,97]. Presence of antibodies to MUC1, a common tumor antigen, showed improved survival for ovarian, gastric, lung and pancreatic cancers [97]. In an ovarian cancer study, TIL B cells were examined and found to have undergone somatic hypermutation, class switch recombination, and oligoclonal expansion. These cells also co-localized with CD8^+^ T cells and the presence of both B and CD8^+^ T cells correlated much more closely to survival than just CD8^+^ infiltrate alone [30]. It is estimated that about 40% of serous ovarian cancers of high grade have significant B cell infiltrate which correlates with survival [98]. The B cells in ovarian cancer are more primed to become APC than any other B cell subtype [98]. B cells themselves have the tools required to directly kill tumor cells via IL-21 mediated granzyme B and IFN-α or TLR-induced TRAIL [30]. However, any B cell activation observed is most likely context dependent. This means that the microenvironment established by the tumor stroma and surrounding cells will dictate whether B cells will incite an immune response or become pro-tumorigenic. 

A decent amount of work has been done in several cancer types to determine which *LTA* variant is associated with increased cancer risk [99,100,101]. There are 4 common single nucleotide polymorphisms (SNPs) in the *LTA* gene, and while the individual functional differences or expression differences have not been elucidated, to date, 3 of the 4 have been implicated in a significantly increased cancer risk [99,100]. These findings are complex and seem to only be valid for specific populations. For example, these variants are associated with increased risk for breast, gastric and lung cancer in Asians [99,100], with colon cancer in Germans and Non-Hodgkin’s Lymphoma in Europeans [99]. Without knowing exactly what the functional relevance of these SNPs are, it is difficult to speculate on the cause for increased risk in specific populations. However, at least in melanoma, hepatocellular and colon cancer, it seems that the presence of LTα or LTβ within the tumor slows growth [101].

CCL19 brought to the tumor site by endothelial progenitor cells (attracted to tumor sites because of ischemic signals) retrovirally infected with a CCL19 vector caused aggressive ovarian tumor growth to slow and reduced lung metastasis by 60% in a mouse model [102]. 

CCL21 injection into pancreatic tumors has been shown to be beneficial by inhibiting tumor growth, decreasing the size of distant metastasis, increasing T cell infiltrate and even enhancing antigen cross-presentation by DC [103]. CCL21 administered intratumorally has even been sufficient to establish TLS within pancreatic cancers [15,103]. However, the expression of both CCR7 and CCL21 in gastric cancer may indicate a poorer prognosis through lymph node metastasis [104] illustrating how context/tumor type-dependent chemokine expression may be with regard to prognosis.

In this regard, although CXCL13 expression has been detected in oral squamous cell carcinoma (OSCC) cells by several groups [105,106,107], expression has not yet been shown to correlate with TLS or immune cell infiltrate in OSCC to date, even though a higher number of immune cells does correlate with longer disease progression intervals [108]. Immune cell populations characterized in primary OSCC samples by Maleki *et al*. include CD4^+^ and CD8^+^ T cells as well as CD20^+^ B cells [108]. Although increased immune cell infiltrate may be a positive prognostic marker in OSCC, the role of CXCL13 in this tumor microenvironment may be a double-edged sword. CXCL13 has been shown to increase the expression and secretion of RANK ligand (RANKL) from the tumor cells themselves [105,106,107], and RANKL has been shown to contribute to secondary lymphoid organ formation [109,110]; yet current data on RANKL expression in OSCC and breast cancer correlate with a more invasive phenotype [108,111,112]. 

### 1.4. Regulators of Tumor-Derived Cytokines and Chemokines that Contribute to TLS Formation

While incoming and resident immune cells no doubt contribute to the pathology of a tumor, the cancer cells themselves may be the first to establish an immunosuppressive microenvironment in order to survive. By understanding some of the most common forms of immunosuppression in cancer, we may begin to unlock the enormous power of our immune systems to eradicate this disease.

As our focus has been on TLS formation and tumor dysregulation of the chemokines and cytokines involved in their development, a cursory search of common transcriptional regulators of *CCL19*, *CCL21*, *CXCL13* and *LTA/B* gene expression may provide insight into potential biomarkers for humoral anti-tumor immunity. The induction of CCL19 and CCL21 expression occurs not only by LTαβ, but also by inflammatory cytokines TNF-α, IL-1β, and lipopolysaccharide (LPS) [26,113]. LTαβ expression is increased after exposure to IL-1β and IL-6 in hepatocytes [114]. These pro-inflammatory signals all channel through the NF-κB pathway. In fact, it has already been shown that *CCL19* contains two NF-κB binding sites in its promoter [113]. Not surprisingly, CXCL13 and LTβ also are transcriptionally regulated by NF-κB signaling [114,115,116]. NF-κB plays a very complex role in cancer. It is normally turned on in response to infections, cellular stress, or by inflammatory cytokines TNF-α or IL-1 [117]. It then upregulates proliferative and pro-survival genes as well as pro-inflammatory genes [117,118]. NF-κB activation is associated with gastric cancer, colon cancer, melanoma and TNF-α-induced EMT in breast cancer [118,119,120]. Mutations of NF-κB itself are relatively rare in solid tumors, indicating that its activation or tumor expression changes are induced by extrinsic signals [120]. The pro-survival NF-κB signals most likely contribute to tumor progression, but its pro-inflammatory pathways may also indirectly inhibit tumor growth. For example, in hepatocellular carcinoma, blockade of NF-κB increases tumor burden [118,120]. The role of NF-κB in cancer appears to be extremely cell-specific and under the influence of the extrinsic environment rather than direct control of the tumor cell. 

In addition to NF-κB, STAT1 and STAT2 have been shown to transcriptionally regulate *CCL19* [113], and STAT3 transcriptionally regulates *CCL21* [121]. STAT (Signal transducer and activators of transcription) molecules are extremely diverse in their function. With regard to tumorigenesis and progression, STAT3 and STAT5 cause increased proliferation, survival, and inhibition of immune responses in several cancer types [122,123]. Inhibition of STAT3 and STAT5 causes apoptosis in pancreatic, breast, renal, colon carcinomas, melanoma [123] and prostate cancer models [124,125]. Increased STAT1 activation, however, is associated with longer overall survival and relapse-free survival in breast cancer [126]. *STAT1* knockout mice have increased tumor incidence, presumably because of a lack of immune surveillance since STAT1 induces IL-12 expression and helps shape a Th1-IFN-γ immune response in collaboration with NF-κB [122]. As is the case in NF-κB signaling, STAT activation and functional consequences are most likely cell-type dependent and while each STAT molecule may have overlapping functions, the individual gene network controlled by each STAT molecule may have major implications for tumor suppression or progression.

Finally, a third major group of key transcriptional regulators of TLS-inducing chemokines is the Interferon Regulatory Factor (IRF) family. *CCL19*, in addition to NF-κB binding sites, also has an interferon (IFN)-sensitive response element (ISRE) consensus sequence within its promoter. The ISRE is the consensus binding site for IRF molecules. CCL19 expression is controlled by at least IRF1, IRF3, IRF7, and IRF9 in the context of particular pathogens in DC [113]. Unpublished work from our lab supports that *CXCL13* has at least four ISRE sites within its promoter and that IRF5 binds to two of them, increasing CXCL13 transcript and protein levels in breast cancer [57]. Additionally, IRF5 increases the transcript levels of *CCL19* and *CCL21* [57].

Although breast cancer has in the past been considered a relatively non-immunogenic cancer, more recent data now provide fairly well-accepted and reproducible findings that the presence of immune cell infiltrate confers a better prognosis [127]. In Soliman *et al*., the authors suggest that the difference in immune infiltrate, and therefore prognosis, is due to the regulation of immune-modulating proteins secreted or controlled by the tumors [127]. In support of this is the fact that a relatively homogeneous group of breast cancer patients (similar age, overall health, and disease type) can be consistently grouped into IR+ or IR-groups indicating that the tumors themselves must be intrinsically different. These data support that the tumors themselves must have differing abilities to elicit or suppress an immune response. Indeed, transcription factors that regulate immune response genes, such as those important for IFN signaling, are often missing in breast cancer [128,129]. To this extent, Bidwell *et al*. found that 540 IFN-regulated genes were consistently suppressed in bone metastases of the 4T1.2 mouse tumor model [128]. Additionally, Bi *et al*. found that IRF5 expression is decreased in approximately 80% of invasive ductal carcinoma samples examined and may regulate a network of cytokines and chemokines involved in the inhibition of metastasis and increased immunogenicity [57,129].

In human lung cancer samples, Li *et al*. examined the relative levels of IRFs expressed and found that IRF5, IRF7 and IRF3 were on average downregulated by 3, 20 and 13-fold, respectively, as compared to normal lung cells [130]. *IFNA* and *IFNB* were also downregulated about 5- to 10-fold, but these numbers varied between samples [130]. Lowered IRF7 expression has also been demonstrated in hepatocellular, gastric, lung and pancreatic cancers while IRF5 downregulation has been shown in breast, hepatocellular and gastric cancer [130].

Interestingly, as was the case with the *LTA* gene, certain SNPs in *IRF5* have been identified in patients with melanoma [131]. A particular SNP in *IRF5* may confer protection from autoimmunity (e.g., SLE) while others are considered “risk haplotypes” for developing SLE. Melanoma patients with the *IRF5* SNP considered protective against SLE were more likely to be non-responsive to immunotherapy treatments. All of the other *IRF5* variants correlated with some level of disease control or regression [131]. Other microarray datasets studied in melanoma implicate *IL-8*, *CXCL13*, *IRF1*, *IRF2* and *IL-12* as possible prognostic markers [56,80]. 

The mechanisms controlling IRF expression and/or activation in tumors is currently not well understood. DNA damage has been shown to upregulate IRF5 expression and induce activation resulting in IRF5-mediated apoptosis [132,133,134]. IRF1 has also been implicated in DNA damage-induced apoptosis [135]. Type I and II IFNs have been shown to upregulate both IRF5 and IRF7 expression [134,136,137,138]. IRF3, on the other hand, may be activated by irregular protein structure or function, based on an *Irf3* knockout mouse model that succumbs to prion diseases more rapidly than the control cohort [139]. Thus, little is known of the mechanism(s) by which expression of IRFs is lost in cancer and whether IR^+^ tumors are directly dependent on IRF expression. Additional work is necessary to understand the activation and function of these transcriptional regulators in IR^+^ tumors.

In summary, the three main transcriptional regulators of TLS formation are NF-κB, STATs, and the IRFs. While they have been most well-studied in immune cell populations, an understanding of their role(s) in normal epithelium is necessary to determine how dysregulation of these factors in cancers lead to immune deficits that tumors acquire to become more invasive. In this simple network of genes required for functional TLS formation (*CCL19*, *CCL20*, *CXCL13*, and *LTA/B*) only a few major transcription factors are thus far implicated. As such, further work in this area is necessary to understand how each of these transcription factors may contribute to the development of solid tumors, as well as TLS formation that will ultimately aid in strengthening a patient's anti-tumor immune response.

## 2. Conclusions

In summary, factors regulating TLS formation in epithelial tissues, such as the chemokines CCL19, CCL21 and CXCL13, and the cytokine LTαβ, most likely also contribute to an anti-tumor immune response in several carcinomas/adenocarcinomas. What remains to be clearly elucidated are (1) whether the tumor cell itself is responsible for expression, or lack thereof, of these critical factors or their upstream regulators (e.g., IRFs, STATs and NF-κB); (2) the immune deficits present in each individual cancer type that result after dysregulation of CCL19, CCL21, CXCL13 and LTαβ expression and/or signaling; and (3) how immunotherapy treatment either alone or in conjunction with current chemotherapy can be used to manipulate the tumor immune environment to re-activate an anti-tumor response.

While it is thought that current chemotherapy treatment allows for the exposure of tumor antigen through tumor necrosis, it is also detrimental to immune cell growth [36,140,141]. Chemotherapy may actually be hindering any fledgling immune response to tumor through its killing of lymphocytes in addition to the tumor target itself. In addition, after noticing that surgical tumor resection often results in metastasis later on, studies on tumor antigen and T cell activation were performed [97,142]. After resection, it was found that tumor antigen load is decreased as well as markers of T cell activation [142] which may offer insight into what is required for a successful immune response. Certainly a high level of tumor antigen would be helpful, as well as the presence of an efficient antigen-presenting, T and B cell activating center, *i*.*e*., a TLS. Current data support that the presence of a TLS augments the tumor immune response.

Enhancing anti-tumor immune responses through cytokine/chemokine administration and tumor antigen vaccination show promise but have yet to lead to consistent, long-term anti-tumor immunity [1,81,140]. In the short term, no validated biomarkers are utilized to predict whether a patient will mount an immune response to tumor [141]. Understanding the mechanisms of immuno-suppression employed by the tumor through dysregulation of TLS-inducing cytokines/chemokines or their transcriptional regulators will allow us to select the most appropriate biomarkers for each cancer type. Categorizing tumors by the expression of either tumor-derived CXCL13, CCL19, CCL21, LTαβ or their key master regulators, such as the IRFs, may allow us to stratify patients more easily into IR^+^ or IR^−^ subtypes. In the long term, understanding how the tumor regulates signals that traffic immune cells, influence their activation, and either elicit or suppress the formation of TLS will allow us to develop immune therapy regiments that spare patients the side effects of non-specific therapy while providing long term tumor immunity.

## References

[1] Neyt K., Perros F., GeurtsvanKessel C.H., Hammad H., Lambrecht B.N. (2012). Tertiary lymphoid organs in infection and autoimmunity. Trends Immunol..

[2] Carragher D.M., Rangel-Moreno J., Randall T.D. (2008). Ectopic lymphoid tissues and local immunity. Sem. Immunol..

[3] Willard-Mack C.L. (2006). Normal structure, function, and histology of lymph nodes. Toxicol. Pathol..

[4] Blum K.S., Pabst R. (2006). Keystones in lymph node development. J. Anat..

[5] Aloisi F., Pujol-Borrell R. (2006). Lymphoid neogenesis in chronic inflammatory diseases. Nat. Rev. Immunol..

[6] Muniz L.R., Pacer M.E., Lira S.A., Furtado G.C. (2011). A critical role for dendritic cells in the formation of lymphatic vessels within tertiary lymphoid structures. J. Immunol..

[7] Rupprecht T.A., Plate A., Adam M., Wick M., Kastenbauer S., Schmidt C., Klein M., Pfister H.W., Koedel U. (2009). The chemokine CXCL13 is a key regulator of B cell recruitment to the cerebrospinal fluid in acute lyme neuroborreliosis. J. Neuroinflamm..

[8] Cassier P.A., Treilleux I., Bachelot T., Ray-Coquard I., Bendriss-Vermare N., Menetrier-Caux C., Tredan O., Goddard-Leon S., Pin J.J., Mignotte H. (2011). Prognostic value of the expression of c-chemokine receptor 6 and 7 and their ligands in non-metastatic breast cancer. BMC Cancer.

[9] Luther S.A., Bidgol A., Hargreaves D.C., Schmidt A., Xu Y., Paniyadi J., Matloubian M., Cyster J.G. (2002). Differing activities of homeostatic chemokines CCL19, CCL21, and CXCL12 in lymphocyte and dendritic cell recruitment and lymphoid neogenesis. J. Immunol..

[10] Ohmatsu H., Sugaya M., Kadono T., Tamaki K. (2007). CXCL13 and CCL21 are expressed in ectopic lymphoid follicles in cutaneous lymphoproliferative disorders. J. Investig. Dermatol..

[11] Mumtaz M., Wagsater D., Lofgren S., Hugander A., Zar N., Dimberg J. (2009). Decreased expression of the chemokine CCL21 in human colorectal adenocarcinomas. Oncol. Rep..

[12] Novak L., Igoucheva O., Cho S., Alexeev V. (2007). Characterization of the CCL21-mediated melanoma-specific immune responses and *in situ* melanoma eradication. Mol. Cancer Ther..

[13] Christopherson K.W., Campbell J.J., Hromas R.A. (2001). Transgenic overexpression of the CC chemokine CCL21 disrupts T-cell migration. Blood.

[14] Kar U.K., Srivastava M.K., Andersson A., Baratelli F., Huang M., Kickhoefer V.A., Dubinett S.M., Rome L.H., Sharma S. (2011). Novel CCL21-vault nanocapsule intratumoral delivery inhibits lung cancer growth. PLoS One.

[15] Ashour A.E., Turnquist H.R., Singh R.K., Talmadge J.E., Solheim J.C. (2007). CCL21-induced immune cell infiltration. Int. Immunopharmacol..

[16] Slight S.R., Rangel-Moreno J., Gopal R., Lin Y., Fallert Junecko B.A., Mehra S., Selman M., Becerril-Villanueva E., Baquera-Heredia J., Pavon L. (2013). CXCR5^+^ T helper cells mediate protective immunity against tuberculosis. J. Clin. Investig..

[17] Cipponi A., Mercier M., Seremet T., Baurain J.F., Theate I., van den Oord J., Stas M., Boon T., Coulie P.G., van Baren N. (2012). Neogenesis of lymphoid structures and antibody responses occur in human melanoma metastases. Cancer Res..

[18] Morita R., Schmitt N., Bentebibel S.E., Ranganathan R., Bourdery L., Zurawski G., Foucat E., Dullaers M., Oh S., Sabzghabaei N. (2011). Human blood CXCR5^+^CD4^+^ T cells are counterparts of t follicular cells and contain specific subsets that differentially support antibody secretion. Immunity.

[19] Chevalier N., Jarrossay D., Ho E., Avery D.T., Ma C.S., Yu D., Sallusto F., Tangye S.G., Mackay C.R. (2011). CXCR5 expressing human central memory CD4 T cells and their relevance for humoral immune responses. J. Immunol..

[20] Singh P., Coskun Z.Z., Goode C., Dean A., Thompson-Snipes L., Darlington G. (2008). Lymphoid neogenesis and immune infiltration in aged liver. Hepatology.

[21] Gunther J., Koczan D., Yang W., Nurnberg G., Repsilber D., Schuberth H.J., Park Z., Maqbool N., Molenaar A., Seyfert H.M. (2009). Assessment of the immune capacity of mammary epithelial cells: Comparison with mammary tissue after challenge with *Escherichia coli*. Vet. Res..

[22] Panse J., Friedrichs K., Marx A., Hildebrandt Y., Luetkens T., Barrels K., Horn C., Stahl T., Cao Y., Milde-Langosch K. (2008). Chemokine CXCL13 is overexpressed in the tumour tissue and in the peripheral blood of breast cancer patients. Br. J. Cancer.

[23] Carlsen H.S., Baekkevold E.S., Johansen F.E., Haraldsen G., Brandtzaeg P. (2002). B cell attracting chemokine 1 (CXCL13) and its receptor CXCR5 are expressed in normal and aberrant gut associated lymphoid tissue. Gut.

[24] Salomonsson S., Larsson P., Tengner P., Mellquist E., Hjelmstrom P., Wahren-Herlenius M. (2002). Expression of the B cell-attracting chemokine CXCL13 in the target organ and autoantibody production in ectopic lymphoid tissue in the chronic inflammatory disease sjogren’s syndrome. Scand. J. Immunol..

[25] Jiang J., Karimi O., Ouburg S., Champion C.I., Khurana A., Liu G., Freed A., Pleijster J., Rozengurt N., Land J.A. (2012). Interruption of CXCL13-CXCR5 axis increases upper genital tract pathology and activation of NKT cells following chlamydial genital infection. PLoS One.

[26] Pickens S.R., Chamberlain N.D., Volin M.V., Pope R.M., Talarico N.E., Mandelin A.M., Shahrara S. (2012). Role of the CCL21 and CCR7 pathways in rheumatoid arthritis angiogenesis. Arthr. Rheum..

[27] Degli-Esposti M.A., Davis-Smith T., Din W.S., Smolak P.J., Goodwin R.G., Smith C.A. (1997). Activation of the lymphotoxin beta receptor by cross-linking induces chemokine production and growth arrest in A375 melanoma cells. J. Immunol..

[28] Coelho F.M., Natale D., Soriano S.F., Hons M., Swoger J., Mayer J., Danuser R., Scandella E., Pieczyk M., Zerwes H.G. (2013). Naive B-cell trafficking is shaped by local chemokine availability and LFA-1-independent stromal interactions. Blood.

[29] Leon B., Ballesteros-Tato A., Browning J.L., Dunn R., Randall T.D., Lund F.E. (2012). Regulation of TH2 development by CXCR5^+^ dendritic cells and lymphotoxin-expressing B cells. Nat. Immunol..

[30] Nelson B.H. (2010). CD20^+^ B cells: The other tumor-infiltrating lymphocytes. J. Immunol..

[31] De Guinoa J.S., Barrio L., Mellado M., Carrasco Y.R. (2011). CXCL13/CXCR5 signaling enhances BCR-triggered B-cell activation by shaping cell dynamics. Blood.

[32] Spaner D., Bahlo A., Medin J., Fowler D. (2011). B Lymphocytes in Cancer Immunology. Experimental and Applied Immunotherapy.

[33] Liu X., Nurieva R.I., Dong C. (2013). Transcriptional regulation of follicular T-helper (TFH) cells. Immunol. Rev..

[34] Smedbakken L.M., Halvorsen B., Daissormont I., Ranheim T., Michelsen A.E., Skjelland M., Sagen E.L., Folkersen L., Krohg-Sorensen K., Russell D. (2012). Increased levels of the homeostatic chemokine CXCL13 in human atherosclerosis-potential role in plaque stabilization. Atherosclerosis.

[35] Coronella-Wood J.A., Hersh E.M. (2003). Naturally occurring B-cell responses to breast cancer. Cancer Immunol. Immunother..

[36] Murphy M.A., O’Leary J.J., Cahill D.J. (2012). Assessment of the humoral immune response to cancer. J. Proteom..

[37] Penn I., Starzl T.E. (1973). Immunosuppression and cancer. Transpl. Proc..

[38] Bergomas F., Grizzi F., Doni A., Pesce S., Laghi L., Allavena P., Mantovani A., Marchesi F. (2011). Tertiary intratumor lymphoid tissue in colo-rectal cancer. Cancers.

[39] Gu-Trantien C., Loi S., Garaud S., Equeter C., Libin M., de Wind A., Ravoet M., le Buanec H., Sibille C., Manfouo-Foutsop G. (2013). CD4^+^ follicular helper T cell infiltration predicts breast cancer survival. J. Clin. Investig..

[40] Coppola D., Nebozhyn M., Khalil F., Dai H., Yeatman T., Loboda A., Mule J.J. (2011). Unique ectopic lymph node-like structures present in human primary colorectal carcinoma are identified by immune gene array profiling. Am. J. Pathol..

[41] Messina J.L., Fenstermacher D.A., Eschrich S., Qu X., Berglund A.E., Lloyd M.C., Schell M.J., Sondak V.K., Weber J.S., Mule J.J. (2012). 12-chemokine gene signature identifies lymph node-like structures in melanoma: Potential for patient selection for immunotherapy?. Sci. Rep..

[42] Gottlin E.B., Bentley R.C., Campa M.J., Pisetsky D.S., Herndon J.E., Patz E.F. (2011). The association of intratumoral germinal centers with early-stage non-small cell lung cancer. J. Thoracic Oncol..

[43] Goc J., Fridman W.H., Sautes-Fridman C., Dieu-Nosjean M.C. (2013). Characteristics of tertiary lymphoid structures in primary cancers. Oncoimmunology.

[44] Denkert C., Loibl S., Noske A., Roller M., Muller B.M., Komor M., Budczies J., Darb-Esfahani S., Kronenwett R., Hanusch C. (2010). Tumor-associated lymphocytes as an independent predictor of response to neoadjuvant chemotherapy in breast cancer. J. Clin. Oncol..

[45] Nzula S., Going J.J., Stott D.I. (2003). Antigen-driven clonal proliferation, somatic hypermutation, and selection of B lymphocytes infiltrating human ductal breast carcinomas. Cancer Res..

[46] Alexe G., Dalgin G.S., Scanfeld D., Tamayo P., Mesirov J.P., DeLisi C., Harris L., Barnard N., Martel M., Levine A.J. (2007). High expression of lymphocyte-associated genes in node-negative HER^2+^ breast cancers correlates with lower recurrence rates. Cancer Res..

[47] Krell J., Frampton A.E., Stebbing J. (2012). The clinical significance of tumor infiltrating lymphoctyes in breast cancer: Does subtype matter?. BMC Cancer.

[48] Coronella J.A., Spier C., Welch M., Trevor K.T., Stopeck A.T., Villar H., Hersh E.M. (2002). Antigen-driven oligoclonal expansion of tumor-infiltrating B cells in infiltrating ductal carcinoma of the breast. J. Immunol..

[49] Mahmoud S.M., Lee A.H., Paish E.C., Macmillan R.D., Ellis I.O., Green A.R. (2012). The prognostic significance of B lymphocytes in invasive carcinoma of the breast. Breast Cancer Res. Treat..

[50] Schmidt M., Bohm D., von Torne C., Steiner E., Puhl A., Pilch H., Lehr H.A., Hengstler J.G., Kolbl H., Gehrmann M. (2008). The humoral immune system has a key prognostic impact in node-negative breast cancer. Cancer Res..

[51] Hansen M.H., Nielsen H., Ditzel H.J. (2001). The tumor-infiltrating B cell response in medullary breast cancer is oligoclonal and directed against the autoantigen actin exposed on the surface of apoptotic cancer cells. Proc. Natl. Acad. Sci. USA.

[52] Pavoni E., Monteriu G., Santapaola D., Petronzelli F., Anastasi A.M., Pelliccia A., D’Alessio V., de Santis R., Minenkova O. (2007). Tumor-infiltrating B lymphocytes as an efficient source of highly specific immunoglobulins recognizing tumor cells. BMC Biotechnol..

[53] Lu H., Goodell V., Disis M.L. (2008). Humoral immunity directed against tumor-associated antigens as potential biomarkers for the early diagnosis of cancer. J. Proteome Res..

[54] Fischer E., Kobold S., Kleber S., Kubuschok B., Braziulis E., Knuth A., Renner C., Wadle A. (2010). Cryptic epitopes induce high-titer humoral immune response in patients with cancer. J. Immunol..

[55] Martinet L., Garrido I., Filleron T., le Guellec S., Bellard E., Fournie J.J., Rochaix P., Girard J.P. (2011). Human solid tumors contain high endothelial venules: Association with T- and B-lymphocyte infiltration and favorable prognosis in breast cancer. Cancer Res..

[56] Liu W., Peng Y., Tobin D.J. (2013). A new 12-gene diagnostic biomarker signature of melanoma revealed by integrated microarray analysis. Peer J..

[57] Pimenta E.M., De S., Feng D., Hall K., Ran S., Barnes  B.J. (2014).

[58] Biswas S.K., Mantovani A. (2010). Macrophage plasticity and interaction with lymphocyte subsets: Cancer as a paradigm. Nat. Immunol..

[59] Wu S., Lu X., Zhang Z.L., Lei P., Hu P., Wang M., Huang B., Xing W., Jiang X.T., Liu H.J. (2011). CC chemokine ligand 21 enhances the immunogenicity of the breast cancer cell line MCF-7 upon assistance of TLR2. Carcinogenesis.

[60] Kim S.J., Shin J.Y., Lee K.D., Bae Y.K., Sung K.W., Nam S.J., Chun K.H. (2012). Microrna let-7a suppresses breast cancer cell migration and invasion through downregulation of C-C chemokine receptor type 7. Breast Cancer Res..

[61] Muller A., Homey B., Soto H., Ge N., Catron D., Buchanan M.E., McClanahan T., Murphy E., Yuan W., Wagner S.N. (2001). Involvement of chemokine receptors in breast cancer metastasis. Nature.

[62] Nguyen-Hoai T., Baldenhofer G., Ahmed M.S., Pham-Duc M., Gries M., Lipp M., Dorken B., Pezzutto A., Westermann J. (2012). CCL19 (ELC) improves Th1-polarized immune responses and protective immunity in a murine HER2/neu DNA vaccination model. J. Gene Med..

[63] Nguyen-Hoai T., Hohn O., Vu M.D., Baldenhofer G., Ahmed M.S.S., Dorken B., Norley S., Lipp M., Pezzutto A., Westermann J. (2012). Ccl19 as an adjuvant for intradermal gene gun immunization in a Her2/neu mouse tumor model: Improved vaccine efficacy and a role for B cells as APC. Cancer Gene Ther..

[64] Marisa L., de Reynies A., Duval A., Selves J., Gaub M.P., Vescovo L., Etienne-Grimaldi M.C., Schiappa R., Guenot D., Ayadi M. (2013). Gene expression classification of colon cancer into molecular subtypes: Characterization, validation, and prognostic value. PLoS Med..

[65] Curtis N.J., Primrose J.N., Thomas G.J., Mirnezami A.H., Ottensmeier C.H. (2012). The adaptive immune response to colorectal cancer: From the laboratory to clinical practice. Eur. J. Surg. Oncol..

[66] Galon J., Costes A., Sanchez-Cabo F., Kirilovsky A., Mlecnik B., Lagorce-Pages C., Tosolini M., Camus M., Berger A., Wind P. (2006). Type, density, and location of immune cells within human colorectal tumors predict clinical outcome. Science.

[67] Camus M., Tosolini M., Mlecnik B., Pages F., Kirilovsky A., Berger A., Costes A., Bindea G., Charoentong P., Bruneval P. (2009). Coordination of intratumoral immune reaction and human colorectal cancer recurrence. Cancer Res..

[68] Yoon H.H., Orrock J.M., Foster N.R., Sargent D.J., Smyrk T.C., Sinicrope F.A. (2012). Prognostic impact of FoxP3^+^ regulatory T cells in relation to CD8^+^ T lymphocyte density in human colon carcinomas. PLoS One.

[69] Xu Y., Xu Q., Yang L., Liu F., Ye X., Wu F., Ni S., Tan C., Cai G., Meng X. (2013). Gene expression analysis of peripheral blood cells reveals toll-like receptor pathway deregulation in colorectal cancer. PLoS One.

[70] Kirman I., Huang E.H., Whelan R.L. (2004). B cell response to tumor antigens is associated with depletion of b progenitors in murine colocarcinoma. Surgery.

[71] Maletzki C., Jahnke A., Ostwald C., Klar E., Prall F., Linnebacher M. (2012). *Ex-vivo* clonally expanded B lymphocytes infiltrating colorectal carcinoma are of mature immunophenotype and produce functional IgG. PLoS One.

[72] Li J., O’Malley M., Sampath P., Kalinski P., Bartlett D.L., Thorne S.H. (2012). Expression of CCL19 from oncolytic vaccinia enhances immunotherapeutic potential while maintaining oncolytic activity. Neoplasia.

[73] Wong K.L., Tai J.J., Wong W.C., Han H., Sem X., Yeap W.H., Kourilsky P., Wong S.C. (2011). Gene expression profiling reveals the defining features of the classical, intermediate, and nonclassical human monocyte subsets. Blood.

[74] Di Caro G., Bergomas F., Grizzi F., Doni A., Bianchi P., Malesci A., Laghi L., Allavena P., Mantovani A., Marchesi F. (2014). Occurrence of tertiary lymphoid tissue is associated to T cell infiltration and predicts better prognosis in early stage colorectal cancers. Clin. Cancer Res..

[75] Jonsson G., Busch C., Knappskog S., Geisler J., Miletic H., Ringner M., Lillehaug J.R., Borg A., Lonning P.E. (2010). Gene expression profiling-based identification of molecular subtypes in stage IV melanomas with different clinical outcome. Clin. Cancer Res..

[76] Dobner B.C., Riechardt A.I., Joussen A.M., Englert S., Bechrakis N.E. (2012). Expression of haematogenous and lymphogenous chemokine receptors and their ligands on uveal melanoma in association with liver metastasis. Acta Ophthalmol..

[77] Charest-Morin X., Pepin R., Gagne-Henley A., Morissette G., Lodge R., Marceau F. (2013). C-C chemokine receptor-7 mediated endocytosis of antibody cargoes into intact cells. Front. Pharmacol..

[78] Dhawan P., Su Y., Thu Y.M., Yu Y., Baugher P., Ellis D.L., Sobolik-Delmaire T., Kelley M., Cheung T.C., Ware C.F. (2008). The lymphotoxin-beta receptor is an upstream activator of NF-κb-mediated transcription in melanoma cells. J. Biol. Chem..

[79] Schrama D., Straten P.T., Fischer W.H., McLellan A.D., Brocker E.B., Reisfeld R.A., Becker J.C. (2001). Targeting of lymphotoxin-alpha to the tumor elicits an efficient immune response associated with induction of peripheral lymphoid-like tissue. Immunity.

[80] Wang E., Miller L.D., Ohnmacht G.A., Mocellin S., Perez-Diez A., Petersen D., Zhao Y., Simon R., Powell J.I., Asaki E. (2002). Prospective molecular profiling of melanoma metastases suggests classifiers of immune responsiveness. Cancer Res..

[81] DiLillo D.J., Yanaba K., Tedder T.F. (2010). B cells are required for optimal CD4^+^ and CD8^+^ T cell tumor immunity: Therapeutic B cell depletion enhances B16 melanoma growth in mice. J. Immunol..

[82] Ladanyi A., Kiss J., Mohos A., Somlai B., Liszkay G., Gilde K., Fejos Z., Gaudi I., Dobos J., Timar J. (2011). Prognostic impact of B-cell density in cutaneous melanoma. Cancer Immunol. Immunother..

[83] Rohrbeck A., Neukirchen J., Rosskopf M., Pardillos G.G., Geddert H., Schwalen A., Gabbert H.E., von Haeseler A., Pitschke G., Schott M. (2008). Gene expression profiling for molecular distinction and characterization of laser captured primary lung cancers. J. Transl. Med..

[84] Kalari K.R., Rossell D., Necela B.M., Asmann Y.W., Nair A., Baheti S., Kachergus J.M., Younkin C.S., Baker T., Carr J.M. (2012). Deep sequence analysis of non-small cell lung cancer: Integrated analysis of gene expression, alternative splicing, and single nucleotide variations in lung adenocarcinomas with and without oncogenic KRAS mutations. Front. Oncol..

[85] Botling J., Edlund K., Lohr M., Hellwig B., Holmberg L., Lambe M., Berglund A., Ekman S., Bergqvist M., Ponten F. (2013). Biomarker discovery in non-small cell lung cancer: Integrating gene expression profiling, meta-analysis, and tissue microarray validation. Clin. Cancer Res..

[86] Roepman P., Jassem J., Smit E.F., Muley T., Niklinski J., van de Velde T., Witteveen A.T., Rzyman W., Floore A., Burgers S. (2009). An immune response enriched 72-gene prognostic profile for early-stage non-small-cell lung cancer. Clin. Cancer Res..

[87] De Chaisemartin L., Goc J., Damotte D., Validire P., Magdeleinat P., Alifano M., Cremer I., Fridman W.H., Sautes-Fridman C., Dieu-Nosjean M.C. (2011). Characterization of chemokines and adhesion molecules associated with T cell presence in tertiary lymphoid structures in human lung cancer. Cancer Res..

[88] Hillinger S., Yang S.C., Batra R.K., Strieter R.M., Weder W., Dubinett S.M., Sharma S. (2006). CCL19 reduces tumour burden in a model of advanced lung cancer. Br. J. Cancer.

[89] Cardell M., Arni S., Yang S.-C., Korom S., Opitz I., Lardinois D., Sharma S., Dubinett S.M., Weder W., Hillinger S. (2006). Combined CCL19/IL-7 treatment eradicates tumors in murine models of lung cancer. AACR Meet. Abstr..

[90] Zhang Q., Sun L., Yin L., Ming J., Zhang S., Luo W., Qiu X. (2013). CCL19/CCR7 upregulates heparanase via specificity protein-1 (SP1) to promote invasion of cell in lung cancer. Tumour Biol..

[91] Riedl K., Baratelli F., Batra R.K., Yang S.C., Luo J., Escuadro B., Figlin R., Strieter R., Sharma S., Dubinett S. (2003). Overexpression of CCL-21/secondary lymphoid tissue chemokine in human dendritic cells augments chemotactic activities for lymphocytes and antigen presenting cells. Mol. Cancer.

[92] Dieu-Nosjean M.C., Antoine M., Danel C., Heudes D., Wislez M., Poulot V., Rabbe N., Laurans L., Tartour E., de Chaisemartin L. (2008). Long-term survival for patients with non-small-cell lung cancer with intratumoral lymphoid structures. J. Clin. Oncol..

[93] Remark R., Alifano M., Cremer I., Lupo A., Dieu-Nosjean M.C., Riquet M., Crozet L., Ouakrim H., Goc J., Cazes A. (2013). Characteristics and clinical impacts of the immune environments in colorectal and renal cell carcinoma lung metastases: Influence of tumor origin. Clin. Cancer Res..

[94] Goc J., Germain C., Vo-Bourgais T.K., Lupo A., Klein C., Knockaert S., de Chaisemartin L., Ouakrim H., Becht E., Alifano M. (2014). Dendritic cells in tumor-associated tertiary lymphoid structures signal a Th1 cytotoxic immune contexture and license the positive prognostic value of infiltrating CD8^+^ T cells. Cancer Res..

[95] Hopewell E.L., Zhao W., Fulp W.J., Bronk C.C., Lopez A.S., Massengill M., Antonia S., Celis E., Haura E.B., Enkemann S.A. (2013). Lung tumor NF-kappaB signaling promotes T cell-mediated immune surveillance. J. Clin. Investig..

[96] Lohr M., Edlund K., Botling J., Hammad S., Hellwig B., Othman A., Berglund A., Lambe M., Holmberg L., Ekman S. (2013). The prognostic relevance of tumour-infiltrating plasma cells and immunoglobulin kappa C indicates an important role of the humoral immune response in non-small cell lung cancer. Cancer Lett..

[97] Reuschenbach M., von Knebel Doeberitz M., Wentzensen N. (2009). A systematic review of humoral immune responses against tumor antigens. Cancer Immunol. Immunother..

[98] Nielsen J.S., Sahota R.A., Milne K., Kost S.E., Nesslinger N.J., Watson P.H., Nelson B.H. (2012). CD20^+^ tumor-infiltrating lymphocytes have an atypical CD27^−^ memory phenotype and together with CD8^+^ T cells promote favorable prognosis in ovarian cancer. Clin. Cancer Res..

[99] Huang Y., Yu X., Wang L., Zhou S., Sun J., Feng N., Nie S., Wu J., Gao F., Fei B. (2013). Four genetic polymorphisms of lymphotoxin-alpha gene and cancer risk: A systematic review and meta-analysis. PLoS One.

[100] Takei K., Ikeda S., Arai T., Tanaka N., Muramatsu M., Sawabe M. (2008). Lymphotoxin-alpha polymorphisms and presence of cancer in 1536 consecutive autopsy cases. BMC Cancer.

[101] Drutskaya M.S., Efimov G.A., Kruglov A.A., Kuprash D.V., Nedospasov S.A. (2010). Tumor necrosis factor, lymphotoxin and cancer. IUBMB Life.

[102] Hamanishi J., Mandai M., Matsumura N., Baba T., Yamaguchi K., Fujii S., Konishi I. (2010). Activated local immunity by CC chemokine ligand 19-transduced embryonic endothelial progenitor cells suppresses metastasis of murine ovarian cancer. Stem Cells.

[103] Turnquist H.R., Lin X., Ashour A.E., Hollingsworth M.A., Singh R.K., Talmadge J.E., Solheim J.C. (2007). CCL21 induces extensive intratumoral immune cell infiltration and specific anti-tumor cellular immunity. Int. J. Oncol..

[104] Hwang T.L., Lee L.Y., Wang C.C., Liang Y., Huang S.F., Wu C.M. (2012). CCL7 and CCL21 overexpression in gastric cancer is associated with lymph node metastasis and poor prognosis. World J. Gastroenterol..

[105] Yuvaraj S., Griffin A.C., Sundaram K., Kirkwood K.L., Norris J.S., Reddy S.V. (2009). A novel function of CXCL13 to stimulate rank ligand expression in oral squamous cell carcinoma cells. Mol. Cancer Res..

[106] Sambandam Y., Sundaram K., Liu A., Kirkwood K.L., Ries W.L., Reddy S.V. (2013). CXCL13 activation of c-Myc induces rank ligand expression in stromal/preosteoblast cells in the oral squamous cell carcinoma tumor-bone microenvironment. Oncogene.

[107] Pandruvada S.N., Yuvaraj S., Liu X., Sundaram K., Shanmugarajan S., Ries W.L., Norris J.S., London S.D., Reddy S.V. (2010). Role of CXC chemokine ligand 13 in oral squamous cell carcinoma associated osteolysis in athymic mice. Int. J. Cancer.

[108] Maleki S., Schlecht N.F., Keller C., Diaz J., Moss J., Prystowsky M.B., Macian F., Brandwein-Gensler M. (2011). Lymphocytic host response to oral squamous cell carcinoma: An adaptive T-cell response at the tumor interface. Head Neck Pathol..

[109] Hess E., Duheron V., Decossas M., Lezot F., Berdal A., Chea S., Golub R., Bosisio M.R., Bridal S.L., Choi Y. (2012). Rankl induces organized lymph node growth by stromal cell proliferation. J. Immunol..

[110] Dougall W.C., Glaccum M., Charrier K., Rohrbach K., Brasel K., de Smedt T., Daro E., Smith J., Tometsko M.E., Maliszewski C.R. (1999). Rank is essential for osteoclast and lymph node development. Genes Dev..

[111] Schramek D., Sigl V., Penninger J.M. (2011). Rankl and rank in sex hormone-induced breast cancer and breast cancer metastasis. Trends Endocrinol. Metab..

[112] Azim H., Azim H.A. (2013). Targeting RANKL in breast cancer: Bone metastasis and beyond. Exp. Rev. Anticancer Ther..

[113] Pietila T.E., Veckman V., Lehtonen A., Lin R., Hiscott J., Julkunen I. (2007). Multiple NF-κb and ifn regulatory factor family transcription factors regulate CCL19 gene expression in human monocyte-derived dendritic cells. J. Immunol..

[114] Subrata L.S., Lowes K.N., Olynyk J.K., Yeoh G.C., Quail E.A., Abraham L.J. (2005). Hepatic expression of the tumor necrosis factor family member lymphotoxin-beta is regulated by interleukin (IL)-6 and IL-1β: Transcriptional control mechanisms in oval cells and hepatoma cell lines. Liver Int..

[115] Britanova L.V., Kuprash D.V. (2009). New putative control elements in the promoter of CXCL13 chemokine gene, a target of alternative NF-κb pathway. Mol. Biol..

[116] Moreth K., Brodbeck R., Babelova A., Gretz N., Spieker T., Zeng-Brouwers J., Pfeilschifter J., Young M.F., Schaefer R.M., Schaefer L. (2010). The proteoglycan biglycan regulates expression of the B cell chemoattractant CXCL13 and aggravates murine lupus nephritis. J. Clin. Investig..

[117] Oeckinghaus A., Ghosh S. (2009). The NF-κb family of transcription factors and its regulation. Cold Spring Harb. Perspect. Biol..

[118] Karin M., Lin A. (2002). NF-κb at the crossroads of life and death. Nat. Immunol..

[119] Li C.W., Xia W., Huo L., Lim S.O., Wu Y., Hsu J.L., Chao C.H., Yamaguchi H., Yang N.K., Ding Q. (2012). Epithelial-mesenchymal transition induced by TNF-alpha requires NF-κb-mediated transcriptional upregulation of twist1. Cancer Res..

[120] Hoesel B., Schmid J.A. (2013). The complexity of NF-κb signaling in inflammation and cancer. Mol. Cancer.

[121] Miyagaki T., Sugaya M., Okochi H., Asano Y., Tada Y., Kadono T., Blauvelt A., Tamaki K., Sato S. (2011). Blocking mapk signaling downregulates CCL21 in lymphatic endothelial cells and impairs contact hypersensitivity responses. J. Investig. Dermatol..

[122] Yu H., Pardoll D., Jove R. (2009). STATs in cancer inflammation and immunity: A leading role for STAT3. Nat. Rev. Cancer.

[123] Furqan M., Akinleye A., Mukhi N., Mittal V., Chen Y., Liu D. (2013). STAT inhibitors for cancer therapy. J. Hematol. Oncol..

[124] Barton B.E., Murphy T.F., Shu P., Huang H.F., Meyenhofer M., Barton A. (2004). Novel single-stranded oligonucleotides that inhibit signal transducer and activator of transcription 3 induce apoptosis *in vitro* and *in vivo* in prostate cancer cell lines. Mol. Cancer Ther..

[125] Barton B.E., Karras J.G., Murphy T.F., Barton A., Huang H.F. (2004). Signal transducer and activator of transcription 3 (STAT3) activation in prostate cancer: Direct STAT3 inhibition induces apoptosis in prostate cancer lines. Mol. Cancer Ther..

[126] Widschwendter A., Tonko-Geymayer S., Welte T., Daxenbichler G., Marth C., Doppler W. (2002). Prognostic significance of signal transducer and activator of transcription 1 activation in breast cancer. Clin. Cancer Res..

[127] Soliman H. (2013). Immunotherapy strategies in the treatment of breast cancer. Cancer Control.

[128] Bidwell B.N., Slaney C.Y., Withana N.P., Forster S., Cao Y., Loi S., Andrews D., Mikeska T., Mangan N.E., Samarajiwa S.A. (2012). Silencing of IRF7 pathways in breast cancer cells promotes bone metastasis through immune escape. Nat. Med..

[129] Bi X., Hameed M., Mirani N., Pimenta E.M., Anari J., Barnes B.J. (2011). Loss of interferon regulatory factor 5 (IRF5) expression in human ductal carcinoma correlates with disease stage and contributes to metastasis. Breast Cancer Res..

[130] Li Q., Tainsky M.A. (2011). Epigenetic silencing of Irf7 and/or Irf5 in lung cancer cells leads to increased sensitivity to oncolytic viruses. PLoS One.

[131] Uccellini L., De Giorgi V., Zhao Y., Tumaini B., Erdenebileg N., Dudley M.E., Tomei S., Bedognetti D., Ascierto M.L., Liu Q. (2012). IRF5 gene polymorphisms in melanoma. J. Transl. Med..

[132] Bi X., Feng D., Korczeniewska J., Alper N., Hu G., Barnes B.J. (2013). Deletion of IRF5 protects hematopoietic stem cells from DNA damage-induced apoptosis and suppresses gamma-irradiation-induced thymic lymphomagenesis. Oncogene.

[133] Hu G., Barnes B.J. (2009). Irf-5 is a mediator of the death receptor-induced apoptotic signaling pathway. J. Biol. Chem..

[134] Hu G., Mancl M.E., Barnes B.J. (2005). Signaling through IFN regulatory factor-5 sensitizes p53-deficient tumors to DNA damage-induced apoptosis and cell death. Cancer Res..

[135] Tamura T., Ishihara M., Lamphier M.S., Tanaka N., Oishi I., Aizawa S., Matsuyama T., Mak T.W., Taki S., Taniguchi T. (1995). An Irf-1-dependent pathway of DNA damage-induced apoptosis in mitogen-activated T lymphocytes. Nature.

[136] Mancl M.E., Hu G., Sangster-Guity N., Olshalsky S.L., Hoops K., Fitzgerald-Bocarsly P., Pitha P.M., Pinder K., Barnes B.J. (2005). Two discrete promoters regulate the alternatively spliced human interferon regulatory factor-5 isoforms. Multiple isoforms with distinct cell type-specific expression, localization, regulation, and function. J. Biol. Chem..

[137] Sato M., Hata N., Asagiri M., Nakaya T., Taniguchi T., Tanaka N. (1998). Positive feedback regulation of type I IFN genes by the IFN-inducible transcription factor IRF-7. FEBS Lett..

[138] Lu R., Moore P.A., Pitha P.M. (2002). Stimulation of IRF-7 gene expression by tumor necrosis factor alpha: Requirement for NF-κB transcription factor and gene accessibility. J. Biol. Chem..

[139] Ishibashi D., Atarashi R., Fuse T., Nakagaki T., Yamaguchi N., Satoh K., Honda K., Nishida N. (2012). Protective role of interferon regulatory factor 3-mediated signaling against prion infection. J. Virol..

[140] Shiao S.L., Ganesan A.P., Rugo H.S., Coussens L.M. (2011). Immune microenvironments in solid tumors: New targets for therapy. Genes Dev..

[141] Fox B.A., Schendel D.J., Butterfield L.H., Aamdal S., Allison J.P., Ascierto P.A., Atkins M.B., Bartunkova J., Bergmann L., Berinstein N. (2011). Defining the critical hurdles in cancer immunotherapy. J. Transl. Med..

[142] Kossenkov A.V., Dawany N., Evans T.L., Kucharczuk J.C., Albelda S.M., Showe L.C., Showe M.K., Vachani A. (2012). Peripheral immune cell gene expression predicts survival of patients with non-small cell lung cancer. PLoS One.

